# Impact of early tumor shrinkage on quality of life in patients treated with first-line cetuximab plus chemotherapy for unresectable metastatic colorectal cancer: results of Phase II QUACK trial

**DOI:** 10.1186/s12885-022-09811-x

**Published:** 2022-06-28

**Authors:** Akira Ooki, Satoshi Morita, Akihito Tsuji, Shigeyoshi Iwamoto, Hiroki Hara, Hiroaki Tanioka, Hironaga Satake, Masato Kataoka, Masahito Kotaka, Yoshinori Kagawa, Masato Nakamura, Tatsushi Shingai, Masashi Ishikawa, Yasuhiro Miyake, Takeshi Suto, Yojiro Hashiguchi, Taichi Yabuno, Masahiko Ando, Junichi Sakamoto, Kensei Yamaguchi

**Affiliations:** 1 Department of Gastroenterological Chemotherapy, Cancer Institute Hospital of Japanese Foundation for Cancer Research, 3-8-31 Ariake, Koto-ku, Tokyo, 135-8550 Japan; 2 Department of Biomedical Statistics and Bioinformatics, Kyoto University, Kyoto, Japan; 3 Department of Medical Oncology, Kagawa University, Kita, Japan; 4 Department of Surgery, Kansai Medical University Kouri Hospital, Neyagawa, Japan; 5 Department of Gastroenterology, Saitama Cancer Center, Saitama, Japan; 6 Department of Clinical Oncology, Kawasaki Medical School, Kurashiki, Japan; 7 Cancer Treatment Center, Kansai Medical University Hospital, Osaka, Japan; 8 Department of Surgery, National Hospital Organization Nagoya Medical Center, Nagoya, Japan; 9 Gastrointestinal Cancer Center, Sano Hospital, Kobe, Japan; 10 Department of Surgery, Kansai Rosai Hospital, Amagasaki, Japan; 11 Aizawa Comprehensive Cancer Center, Aizawa Hospital, Matsumoto, Japan; 12 Department of Surgery, Osaka Saiseikai Senri Hospital, Suita, Japan; 13 Department of Surgery, Shikoku Central Hospital, Shikokuchuo, Japan; 14 Department of Surgery, Osaka Minato Central Hospital, Osaka, Japan; 15 Department of Surgery, Yamagata Prefectural Central Hospital, Yamagata, Japan; 16 Department of Surgery, Teikyo University School of Medicine, Tokyo, Japan; 17 Department of Surgery, Yokohama Municipal Citizen’s Hospital, Yokohama, Japan; 18 Department of Advanced Medicine, Nagoya University Hospital, Nagoya, Japan; 19 Tokai Central Hospital, Kakamigahara, Japan

**Keywords:** Early tumor shrinkage, Patient-reported outcome, Colorectal cancer, Cetuximab

## Abstract

**Purpose:**

Although early tumor shrinkage (ETS) is a predictor of improved overall survival (OS), the association between ETS and health-related quality of life (HRQOL) remains unclear for patients with metastatic colorectal cancer (mCRC) treated with first-line cetuximab plus chemotherapy.

**Methods:**

The data were collected from a prospective trial that assessed HRQOL using the EORTC QLQ-C30. The impact of ETS on HRQOL was estimated using a linear mixed-effects model for repeated measures.

**Results:**

ETS was achieved in 82 (64.1%) of 128 mCRC patients treated with first-line cetuximab plus chemotherapy, and these patients had a significantly longer OS than those without ETS (HR, 0.38; 95% CI, 0.20–0.72; P = .002). Asymptomatic patients with ETS had a favorable OS, while symptomatic patients without ETS had a worse OS (2-year OS rates, 77.8% vs. 42.5%). Symptomatic patients with ETS had similar outcomes as asymptomatic patients without ETS (2-year OS rates, 64.1% vs. 67.0%). For symptomatic patients, ETS was associated with improved HRQOL scores between baseline and 8 weeks: the mean changes for patients with and without ETS were 5.86 and -4.94 for global health status (GHS)/QOL, 26.73 and 3.79 for physical functioning, and 13.58 and -3.10 for social functioning, respectively. The improved HRQOL was comparable to that of asymptomatic patients without ETS. For asymptomatic patients, ETS showed a decreased deterioration in HRQOL.

**Conclusion:**

Our findings highlight the importance of ETS for HRQOL and prognostic estimates, and assessing ETS may provide clinically useful information for physicians and patients to make more informed decisions.

**Supplementary Information:**

The online version contains supplementary material available at 10.1186/s12885-022-09811-x.

## Introduction

Colorectal cancer (CRC) risk is predominantly driven by environmental factors [1]. CRC has the third highest incidence among cancers and is the second leading cause of cancer-related deaths worldwide; thus, it remains a major clinical challenge [2]. Almost one-fourth of CRC patients have metastatic disease at the time of diagnosis [3, 4]. Despite advances in the treatment of patients with metastatic CRC (mCRC), treatment at this stage is essentially palliative [4, 5]. Accordingly, in addition to the prevention of tumor progression and the prolongation of survival, both improving tumor-related symptoms and maintaining health-related quality of life (HRQOL) should be taken into consideration when planning treatment [5]. In fact, cancer patients tend to request more information on not only survival estimates but also HRQOL [6]. Therefore, an assessment of the impacts of both the treatment and disease on HRQOL is urgently needed.

HRQOL comprises mental, physical, and social well-being, all of which are affected by the tumor burden and/or adverse events [7, 8]. It is difficult for physicians to accurately determine how a patient feels or functions with respect to a health condition because of their dependence on the patient’s perception [9–11]. In fact, the U.S. The Food and Drug Administration stated that some treatment effects are known only to the patient, and such information can be lost when the patient’s perspective is filtered through a physician’s evaluation of the patient’s responses to clinical interview questions [12]. A patient-reported outcome (PRO) is defined as any report about a health condition and treatment obtained directly from the patient, without interpretation of the patient’s response by a physician or anyone else, via standardized questionnaires designed to measure a conceptual framework of HRQOL, including symptoms, satisfaction, or functioning [12, 13]. Thus, PROs are an umbrella term encompassing patient self-reported outcomes related to a patient’s health status and perceptions, and PROs can bridge the considerable gap in reported HRQOL between patients and physicians [9], representing an effective approach to improve the quality of care for patients.

Cetuximab, an antibody targeting epidermal growth factor receptor (EGFR), has been demonstrated to have promising efficacy when combined with chemotherapy as a first-line treatment for left-sided and *RAS* wild-type mCRC [14–17]. Of note, there is a growing body of evidence demonstrating that cetuximab plus chemotherapy more effectively promotes early tumor shrinkage (ETS) than chemotherapy alone or bevacizumab plus chemotherapy and that ETS is associated with long-term OS, possibly by achieving a maximal depth of response [16, 18]. As HRQOL is a major concern for mCRC patients because their tumor burden often results in them being symptomatic at the time of diagnosis [19, 20], ETS may rapidly improve HRQOL for symptomatic patients at baseline. However, the clinical impact of ETS on HRQOL has not yet been determined in mCRC patients treated with first-line cetuximab plus chemotherapy.

The QUACK study was prospectively performed to assess the HRQOL of mCRC patients treated with cetuximab plus chemotherapy using a PRO assessment tool [9, 19, 21]. The aim of the present study was to assess the association of ETS with HRQOL and prognostic outcomes according to the baseline symptom status by performing a post hoc analysis of the QUACK trial data. Our findings will provide additional relevant information that may help both patients and physicians make more informed clinical decisions.

## Patients and Methods

### Study design and treatment

The QUACK study was a prospective, multicenter, phase II study that assessed the associations of QOL with adverse events and treatment efficacy for mCRC patients treated with first-line cetuximab plus standard chemotherapy (FOLFOX or FOLFIRI). Detailed information on the study design has been provided previously [22]. In total, 149 patients with *KRAS* wild-type mCRC were enrolled from 49 institutions between July 2013 and April 2015, of which 140 patients received cetuximab plus chemotherapy at least once. The chemotherapy regimen (FOLFOX or FOLFIRI) was selected according to the treating physician’s discretion and institutional guidelines. The primary results have already been published elsewhere [21].

This study was registered with the University Hospital Medical Information Network (UMIN) Clinical Trial Registry (UMIN000010985) on July 19, 2013 and was conducted in accordance with the Declaration of Helsinki and the Ethics Guidelines for Clinical Research issued by the Ministry of Health, Labor, and Welfare in Japan. The study protocol was approved by the institutional review board, and written informed consent was obtained from all patients before registration.

### Treatment efficacy

Radiologic assessments were performed using computed tomography at baseline and every 8 weeks during the treatment period. The investigator at each institution assessed the tumor response based on the Response Evaluation Criteria in Solid Tumors (RECIST) version 1.1. The overall response rate (ORR) was calculated as the proportion of patients with a complete response or a partial response according to the RECIST criteria. ETS was defined as a relative reduction of ≥ 20% in the sum of the longest diameters of target lesions between the values at baseline and 8 weeks [18]. The ETS cutoff value of ≥ 20% was determined in a time-dependent receiver operating characteristic curve analysis [18], and the 8-week time point was expected to minimize the influence of early study termination due to the first radiologic assessment.

Progression-free survival (PFS) was defined as the time interval between registration and the date of tumor progression or death. Time to treatment failure (TTF) was defined as the time interval between registration and the date of treatment discontinuation for any reason, including treatment toxicity, tumor progression, patient withdrawal, or death. Overall survival (OS) was defined as the time from registration to the date of death from any cause.

### HRQOL and symptom assessments

Because the QUACK study was specifically designed to assess the associations of HRQOL with adverse events and treatment efficacy, HRQOL assessments were typically performed at baseline and after 2, 4, 8, 16, and 24 weeks. The survey sheets were collected at registration and after 4, 8, 16, and 24 weeks, and these surveys also included the patients’ assessments of safety, compliance with treatment, and treatment efficacy.

HRQOL was evaluated using the European Organization for Research and Treatment of Cancer Quality of Life Questionnaire C30 (EORTC QLQ-C30) version 3.0, which is a valid and reliable PRO instrument for assessing HRQOL in cancer settings [23, 24]. The EORTC QLQ-C30 questionnaire is composed of both single- and multi-item scales, including a global health status (GHS)/QOL scale, five functional scales (role, physical, cognitive, emotional, and social), and eight symptom scales (appetite loss, pain, diarrhea, constipation, dyspnea, fatigue, insomnia, and nausea/vomiting) [23]. The observed raw data were standardized through a linear transformation, and the scores ranged from 0 to 100, with a higher score indicating better levels of GHS/QOL and functioning [25]. The symptom scales have four response categories for each question (“very much,” “quite a bit,” “a little,” and “not at all”). Patients were defined as symptomatic when they reported “very much” or “quite a bit” to at least one of the symptom questions at baseline and as asymptomatic when they reported only “a little” or “not at all” to all eight symptom scales [21, 26].

### Statistical analysis

All analyses performed in this study included eligible patients who underwent any intervention after registration and who answered the HRQOL questionnaire at both baseline and at least once postbaseline. To assess the impact of ETS on HRQOL, the association of ETS with changes in the EORTC QLQ-C30 scores from baseline throughout the observation period of 24 weeks was analyzed using a linear mixed-effects model for repeated measures, with the intercept and slope for the study week treated as random effects to estimate the least squares means of the change from baseline. A statistical analysis was also performed to evaluate the association between ETS and HRQOL according to the baseline symptom status.

The distribution of the prognostic outcomes was estimated using the Kaplan–Meier method, and the log-rank test was used to compare the distribution between the populations. The Cox proportional hazard model was used to analyze the association between ETS and the time-to-event endpoints, for which the adjusted hazard ratios (HRs) and the 95% confidence intervals (CIs) were calculated.

The continuous data variables were expressed as the mean ± the standard error of the mean (SEM), and they were compared using a two-tailed Student’s t test. Fisher’s exact test was used for categorical variables. The JMP 14 software package (SAS Institute, Cary, NC, USA) was used to conduct all statistical analyses.

## Results

### Clinicopathological characteristics associated with ets following cetuximab plus chemotherapy

**Fig. 1:**
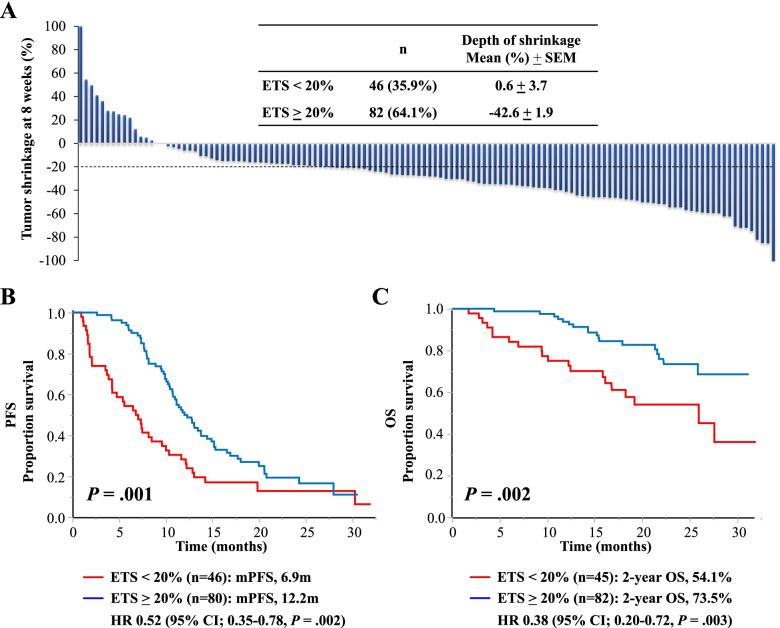
Association between ETS and prognostic outcomes in 128 mCRC patients treated with cetuximab plus chemotherapy. **A)** Waterfall plots of individual patient tumor shrinkage at 8 weeks from the initiation of treatment. **B)** Kaplan–Meier curves of PFS according to ETS status. **C)** Kaplan–Meier curves of OS according to ETS status

This study analyzed a dataset collected from 128 of 140 mCRC patients treated with first-line cetuximab plus chemotherapy in the prospective QUACK study (Table 1). The median age was 66 years (range, 27–89 years), 87 (68.0%) were male, and 107 (83.6%) had an Eastern Cooperative Oncology Group Performance Status (ECOG PS) of 0. At baseline, 51 patients (39.8%) were symptomatic, reporting “very much” or “quite a bit” for at least one of the eight symptom items (i.e., appetite loss, pain, diarrhea, constipation, dyspnea, fatigue, insomnia, and nausea/vomiting) on the EORTC QLQ-C30 questionnaire. Although ETS may depend on the location and size of metastases [27], ETS was achieved in 82 (64.1%) of 128 patients treated with first-line cetuximab plus chemotherapy (Fig. 1A), consistent with previous reports, including an ETS rate of 61.5–80% [16, 18, 28–31]. Baseline clinicopathological characteristics were well balanced between patients with and without ETS, except for differentiation (Table 1). ETS was associated with a higher ORR (76.8% vs. 21.7%; *P* < .001). The rate of surgical resection of metastases was significantly higher among patients with ETS than among those without ETS (28.6% vs. 11.4%; *P* = .031).

**Table 1:** Correlation of clinicopathologic characteristics with ETS in 128 metastatic colorectal cancer patients

Variables		ETS < 20%		ETS **>** 20%		***P*** value
	Total No.	No.	(%)	No.	(%)	
Total No.	128	46	(35.9)	82	(64.1)	
Age (years)						
Mean ± SEM		64.6 ± 1.5		65.3 ± 1.2		NS (.689)^a^
< 70	83	28	(60.9)	55	(67.1)	NS (.481)
> 70	45	18	(39.1)	27	(32.9)	
Gender						
Male	87	32	(69.6)	55	(67.1)	NS (.772)
Female	41	14	(30.4)	27	(32.9)	
ECOG PS						
PS0	107	38	(82.6)	69	(84.1)	NS (.822)
PS1 or PS2	21	8	(17.4)	13	(15.9)	
EORTC QLQ-C30 (Mean ± SEM)						
GHS/QoL	128	60.1 ± 3.3		61.8 ± 2.5		NS (.688)^a^
Social functioning	127	83.7 ± 3.1		82.1 ± 2.3		NS (.681)^a^
Physical functioning	127	80.4 ± 2.9		87.5 ± 2.2		NS (.051)^a^
Role functioning	123	83.0 ± 3.8		87.4 ± 2.9		NS (.359)^a^
Cognitive functioning	127	81.9 ± 2.7		84.6 ± 2.0		NS (.422)^a^
Emotional functioning	128	78.1 ± 2.6		79.1 ± 2.0		NS (.779)^a^
Tumor location						
Colon	83	26	(56.5)	57	(69.5)	NS (.140)^a^
Rectum	45	20	(43.5)	25	(30.5)	
Differentiation						
well/mode	121	41	(89.1)	80	(97.6)	.044
poor	7	5	(10.9)	2	(2.4)	
Number of metastatic lesions						
1	46	18	(39.1)	28	(34.1)	NS (.573)
≥ 2	82	28	(60.9)	54	(65.9)	
Serum CEA (ng/ml)						
< 5	24	10	(22.2)	14	(17.9)	NS (.565)
≥ 5	99	35	(77.8)	64	(82.1)	
Primary tumor						
Absence	85	31	(67.4)	54	(66.7)	NS (.934)
Presence	42	15	(32.6)	27	(33.3)	
Chemotherapy backbone						
mFOLFOX6	82	27	(58.7)	55	(67.1)	NS (.343)
FOLFIRI	46	19	(41.3)	27	(32.9)	
Tumor response						
CR/PR	73	10	(21.7)	63	(76.8)	< .001
SD	43	24	(52.2)	19	(23.2)	
PD	12	12	(26.1)	0	(0)	
Conversion surgery						
Absence	89	39	(88.6)	50	(71.4)	.031
Presence	25	5	(11.4)	20	(28.6)	
Second line						
Absence	29	13	(29.5)	16	(23.2)	NS (.451)
Presence	84	31	(70.5)	53	(76.8)	

*Abbreviations*: *ETS* early tumor shrinkage, *ECOG PS* Eastern Cooperative Oncology Group Performance Status, *EORTC QLQ-C30* European Organization for Research and Treatment of Cancer Quality of Life Questionnaire Core 30; *GHS/QoL* global health status/quality of life, *CR* complete response, *PR* partial response, *SD* stable disease, *PD* progressive disease

*NS* not significant, *SEM* standard error of the mean.

^a^ unpaired Student’s t test; the remaining variables, Fisher's exact test

### Association of ETS with prognosis according to the symptomatic status at baseline

At the data cutoff point on April 20, 2016, 103 and 39 events were observed in relation to PFS and OS, respectively. The median follow-up time was 18.1 months (95% CI, 17.2–19.7), and the median PFS was 10.8 months (95% CI, 9.6–12.1). The median OS was not reached at the data cutoff point, and the 2-year estimated OS rate was 66.7%. Patients with ETS had a significantly longer PFS than those without ETS: the median PFS values were 12.2 months for patients with ETS and 6.9 months for those without ETS (log-rank test, *P* = .001, HR, 0.52; 95% CI, 0.35–0.78; Fig. 1B). Achieving ETS was also associated with a better OS (2-year OS rates, 73.5% vs. 54.1%; log-rank test, *P* = .002; HR, 0.38; 95% CI, 0.20–0.72; Fig. 1C) and TTF (median TTF, 7.7 vs. 4.1 months; log-rank test, *P* < .001; HR, 0.48; 95% CI, 0.33–0.70; Fig. S1). In addition, higher ETS values were significantly associated with longer times for PFS, OS, and TTF when ETS was analyzed as a continuous variable (i.e., the percentage of tumor shrinkage at 8 weeks) (Fig. S2).

**Fig. 2:**
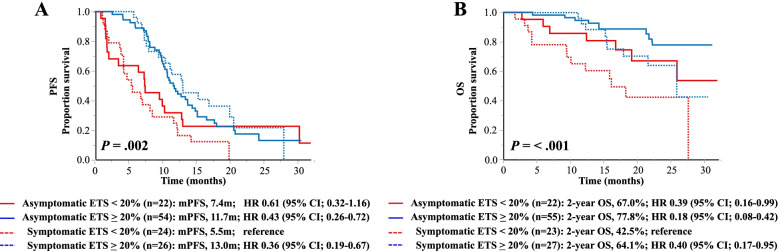
Kaplan–Meier curves of **A)** PFS and **B)** OS according to ETS status based on symptoms reported by patients at baseline using the symptom scales of the EORTC QLQ-C30 questionnaire. Compared with symptomatic patients without ETS as a reference, the HR and 95% CI were calculated for each population

We previously found that the presence of baseline patient-reported symptoms, as an independent predictor, was associated with a significantly worse OS in mCRC patients treated with cetuximab plus chemotherapy [19]. Because ETS was associated with a better OS, the association of ETS with prognostic outcomes based on the baseline symptom status was evaluated. ETS had a significant impact on PFS irrespective of the baseline symptom status: the median PFS was 13.0 months for symptomatic patients with ETS and 11.7 months for asymptomatic patients with ETS, while it was 5.5 months for symptomatic patients without ETS and 7.4 months for asymptomatic patients without ETS. Compared with symptomatic patients without ETS as a reference, the HRs were 0.36 (95% CI: 0.19–0.67) for symptomatic patients with ETS, 0.43 (95% CI: 0.26–0.72) for asymptomatic patients with ETS, and 0.61 (95% CI: 0.32–1.16) for asymptomatic patients without ETS (Fig. 2A). Next, survival estimates of OS were determined according to the baseline symptom status (Fig. 2B). In terms of the 2-year OS rates, asymptomatic patients with ETS had the most favorable outcomes (77.8%), while symptomatic patients without ETS had the worst outcomes (42.5%). Symptomatic patients who achieved ETS had similar prognostic characteristics as asymptomatic patients without ETS (64.1% vs. 67.0%). Compared with symptomatic patients who did not achieve ETS as a reference, the HRs were 0.18 (95% CI: 0.08–0.42) for asymptomatic patients with ETS, 0.40 (95% CI: 0.17–0.95) for symptomatic patients with ETS, and 0.39 (95% CI: 0.16–0.99) for asymptomatic patients without ETS.

### Association between ETS and HRQOL according to the symptomatic status at baseline

**Fig. 3:**
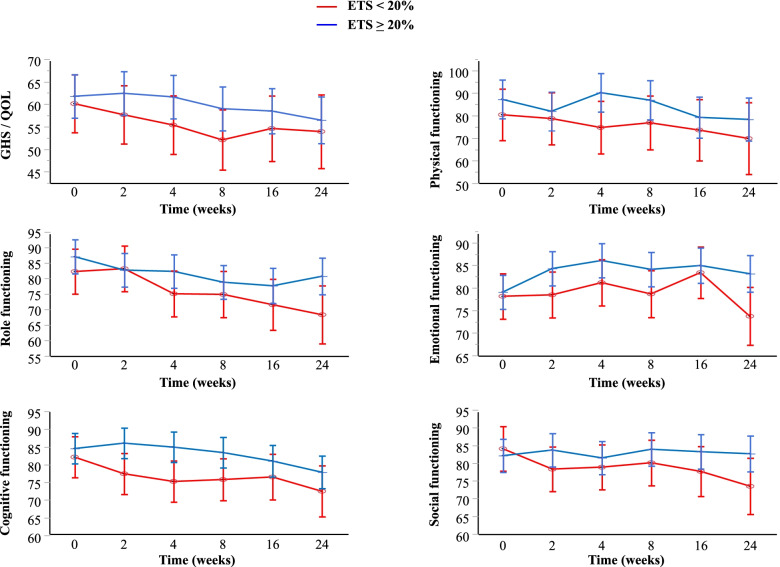
Association of ETS with HRQOL from baseline throughout the study period of 24 weeks using a linear mixed-effects model for repeated measures. The least squares means of the score at each time point were determined for the GHS/QOL and five functional (physical, role, emotional, cognitive, social) scales

Although GHS/QOL and five functional (physical, social, emotional, role, and cognitive) scales are included among the HRQOL scales in the EORTC QLQ-C30 questionnaire, the association between ETS and each scale remains unclear. The impact of ETS on the changes in HRQOL scores from baseline was estimated using a linear mixed-effects model for repeated measures (Fig. 3). Less deterioration in all HRQOL scales was observed among patients with ETS than among those without ETS throughout the 24-week study period: the respective estimated mean score changes from baseline to 8 weeks were -2.78 vs. -8.05 for GHS/QOL, 5.04 vs. 0.51 for emotional functioning, -1.13 vs. -6.34 for cognitive functioning, -0.35 vs. -3.55 for physical functioning, 1.81 vs. -3.97 for social functioning, and -8.23 vs. -7.40 for role functioning.

**Fig. 4:**
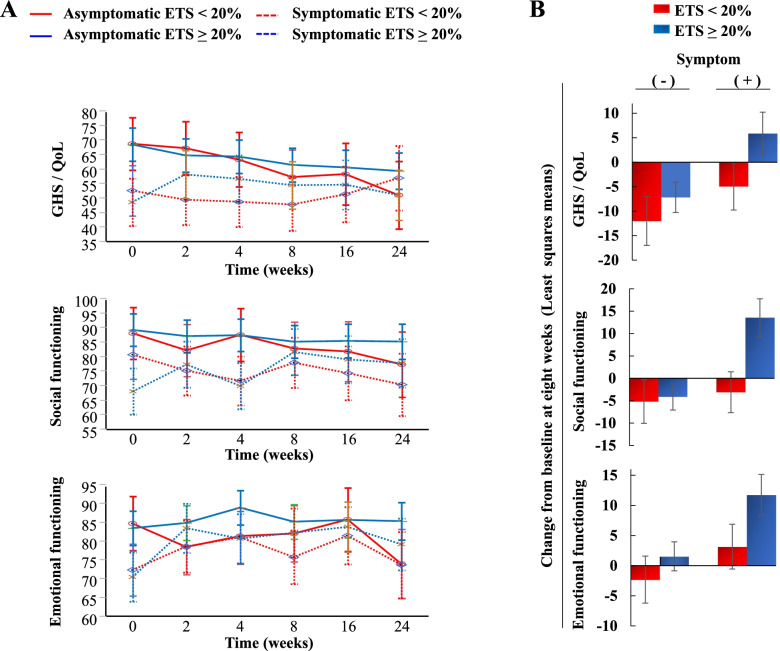
Association of ETS with HRQOL according to the baseline symptom status. **A)** The least squares means of the score at each time point throughout the study period for GHS/QOL, social functioning, and emotional functioning. **B)** The least squares means of the score change from baseline to 8 weeks after initiation of treatment for GHS/QOL, social functioning, and emotional functioning

To evaluate the association between ETS and HRQOL based on the baseline symptom status, changes in HRQOL scores according to ETS were assessed in subgroups of patients with and without symptoms at baseline (Fig. 4 and S3). For symptomatic patients, ETS was associated with improved HRQOL scores from baseline to 8 weeks after initiation of treatment: the respective estimated mean score changes for patients with and without ETS were 5.86 and -4.94 for GHS/QOL, 11.73 and 3.15 for emotional functioning, 6.79 and -2.24 for cognitive functioning, 26.73 and 3.79 for physical functioning, 13.58 and -3.10 for social functioning, and 6.94 and 2.19 for role functioning. For asymptomatic patients, ETS was associated with the lowest degree of deterioration in all HRQOL scales throughout the study period. Of note, symptomatic patients with ETS had similar characteristics as asymptomatic patients without ETS in relation to HRQOL throughout the study period.

## Discussion

HRQOL provides a reasonably comprehensive picture of a patient’s experience during their disease and treatment [32]. PROs are becoming increasingly crucial for capturing the subjective aspects of a patient’s HRQOL because of the substantial disagreement between physician and patient assessments of a patient’s health status and perceptions [9, 33]. HRQOL may be even more relevant for mCRC patients because the aim of treatment is generally palliative rather than curative [5] and because they have many tumor-related symptoms, such as constipation, pain, fatigue, and appetite loss [19, 20]. Consequently, consideration should be given to not only the prognosis but also the rapid improvement of HRQOL when planning treatments for symptomatic patients. However, the lack of this invaluable information is among the main problems faced during the treatment of mCRC. Here, we analyzed the clinical impacts of ETS on HRQOL using a PRO assessment tool and on prognostic relevance in baseline symptomatic mCRC patients treated with first-line cetuximab plus chemotherapy.

ETS was associated with maintaining HRQOL in an exploratory analysis of the phase III ABSOLUTE trial that assessed the treatment efficacy of nanoparticle albumin-bound paclitaxel vs. solvent-based paclitaxel in second-line chemotherapy for advanced gastric cancer [34]. We previously reported that response to treatment was associated with a clinically meaningful improvement in HRQOL for symptomatic mCRC patients [21]. Therefore, we hypothesized that ETS may result in faster symptom relief because of the rapid reduction of the tumor burden for mCRC patients with baseline symptoms. However, there have been few in-depth assessments of the effects of ETS on the HRQOL of mCRC patients according to their baseline symptom status [35]. This exploratory analysis used the EORTC questionnaire and found that ETS was associated with less deterioration of GHS/QOL and functioning. In a retrospective analysis of data from three trials of first-line chemotherapy plus the anti-EGFR antibody panitumumab in *RAS*-wild-type mCRC, the onset of new tumor-related symptoms, defined as new opiate use, weight loss, anemia, asthenia, and decline in ECOG PS, was delayed by achieving ETS, regardless of the treatment regimen received [36]. These findings suggest that HRQOL may remain relatively constant in mCRC patients with ETS. Of note, for symptomatic patients at baseline, ETS was associated with an improved HRQOL, and their status was comparable to that of asymptomatic patients without ETS at 8 weeks. Thus, achieving ETS may be of particular benefit to mCRC patients with baseline symptoms in terms of HRQOL. Considering the high proportion of ETS in patients receiving FOLFOXIRI plus bevacizumab versus FOLFIRI plus bevacizumab [37], it is also important to evaluate whether ETS could be a predictor of PROs even in mCRC patients treated with bevacizumab plus chemotherapy. Future studies are needed to clarify the benefit of ETS for HRQOL.

In clinical trials, tumor response based on the RECIST criteria is defined as at least a 30% reduction in the tumor, but this definition does not consider the timing of the response. Importantly, the objective response rate is not a reliable surrogate for PFS or OS [38]. On the other hand, ETS is defined as a minimum tumor reduction of 20% 6–8 weeks after the initiation of treatment, and it has been found to be associated with favorable prognostic outcomes in post hoc analyses in several trials, independent of the agents used and cancer types [16, 18, 28, 29, 31, 36, 37, 39–46]. We confirmed that ETS was significantly associated with prolonged PFS, OS, and TTF. Although the presence of baseline patient-reported symptoms has been reported as an independent prognostic factor in mCRC patients [19, 47], ETS has been associated with an improved OS even for symptomatic patients, who had similar outcomes as asymptomatic patients without ETS. Consistent with previous reports [39, 48], ETS was associated with successful conversion surgery. Collectively, ETS may be a hallmark feature of sensitivity to treatment and will offer several clinical advantages, including serving as an early predictor of treatment efficacy, guiding treatment strategies regarding surgical resection for potentially resectable disease, and providing a means for rapidly improving HRQOL.

The phase III CRYSTAL study reported a significant survival advantage of first-line cetuximab plus chemotherapy over chemotherapy alone in the treatment of *KRAS* wild-type mCRC [49]. Subsequently, in two phase III trials, FIRE-3/AIO KRK0306 and CALGB/SWOG 80405, evaluating the first-line treatment efficacy of cetuximab versus an antibody targeting vascular endothelial growth factor (bevacizumab) in combination with chemotherapy, the median OS was significantly better in the cetuximab plus chemotherapy group than in the bevacizumab plus chemotherapy group for patients with left-sided and *RAS*-wild type mCRC [16, 17]. Cetuximab maintenance treatment also tended to have a superior survival benefit when compared with bevacizumab maintenance in *RAS*-wild type mCRC [50]. Of note, the rate of ETS was markedly higher in the cetuximab plus chemotherapy group than in the chemotherapy alone group in the CRYSTAL trial (61.5% vs. 49.1%) [18] or in the bevacizumab plus chemotherapy group in the FIRE-3/AIO KRK0306 trial (68.2% vs. 49.1%) [16], consistent with the findings of this study (64.1% for cetuximab plus chemotherapy). In addition, our previous findings indicated that there was no deterioration in HRQOL following the addition of cetuximab to chemotherapy in the CRYSTAL study [51] and that the toxicity profiles and effects on PFS and ORR were similar for patients receiving cetuximab plus chemotherapy irrespective of their baseline symptom status in the QUACK study [19]. Taken together, cetuximab plus chemotherapy can notably accelerate ETS, suggesting that it may be the most preferred first-line regimen for left-sided and *RAS* wild-type mCRC patients, especially for those with baseline symptoms, from the point of view of HRQOL, prognosis, and safety.

To our knowledge, this is the first study to report the clinical impacts of ETS on HRQOL according to the baseline symptom status in mCRC patients treated with first-line cetuximab plus chemotherapy. However, the presented results are limited by the exploratory retrospective nature of the analysis and the relatively small sample size. In addition, the interpretation of the findings is limited by the design of comparisons between subgroups of patients treated with cetuximab plus chemotherapy because of the single-arm study. Because the differences in the efficacy of cetuximab according to the location of the primary tumor had not yet been demonstrated when this study began [14], there were no data on tumor sidedness. On the other hand, the main strengths of this study are the use of data from questionnaires with high completion rates, the prospective design for studying HRQOL, and the use of a well-established global PRO assessment tool [21].

In conclusion, ETS may be useful not only as an early-on-treatment predictor of treatment efficacy but also to rapidly improve HRQOL for symptomatic patients, which will facilitate patient-centered care in clinical practice.

## Supplementary Information

**Additional file 1.**

**Additional file 2.**

**Additional file 3.**

## Data Availability

All data analyzed during this study are included in this published article and its supplementary information files.
