# Supplementary material for: Impact of early tumor shrinkage on quality of life in patients treated with first-line cetuximab plus chemotherapy for unresectable metastatic colorectal cancer: results of Phase II QUACK trial
Source: BMC Cancer. 2022 Jun 28;22:711. doi: 10.1186/s12885-022-09811-x (PMC9238042; doi:10.1186/s12885-022-09811-x)
Supplement: Supplementary file 1 — Additional file 1. [file 12885_2022_9811_MOESM1_ESM.pptx]

## Slide 1
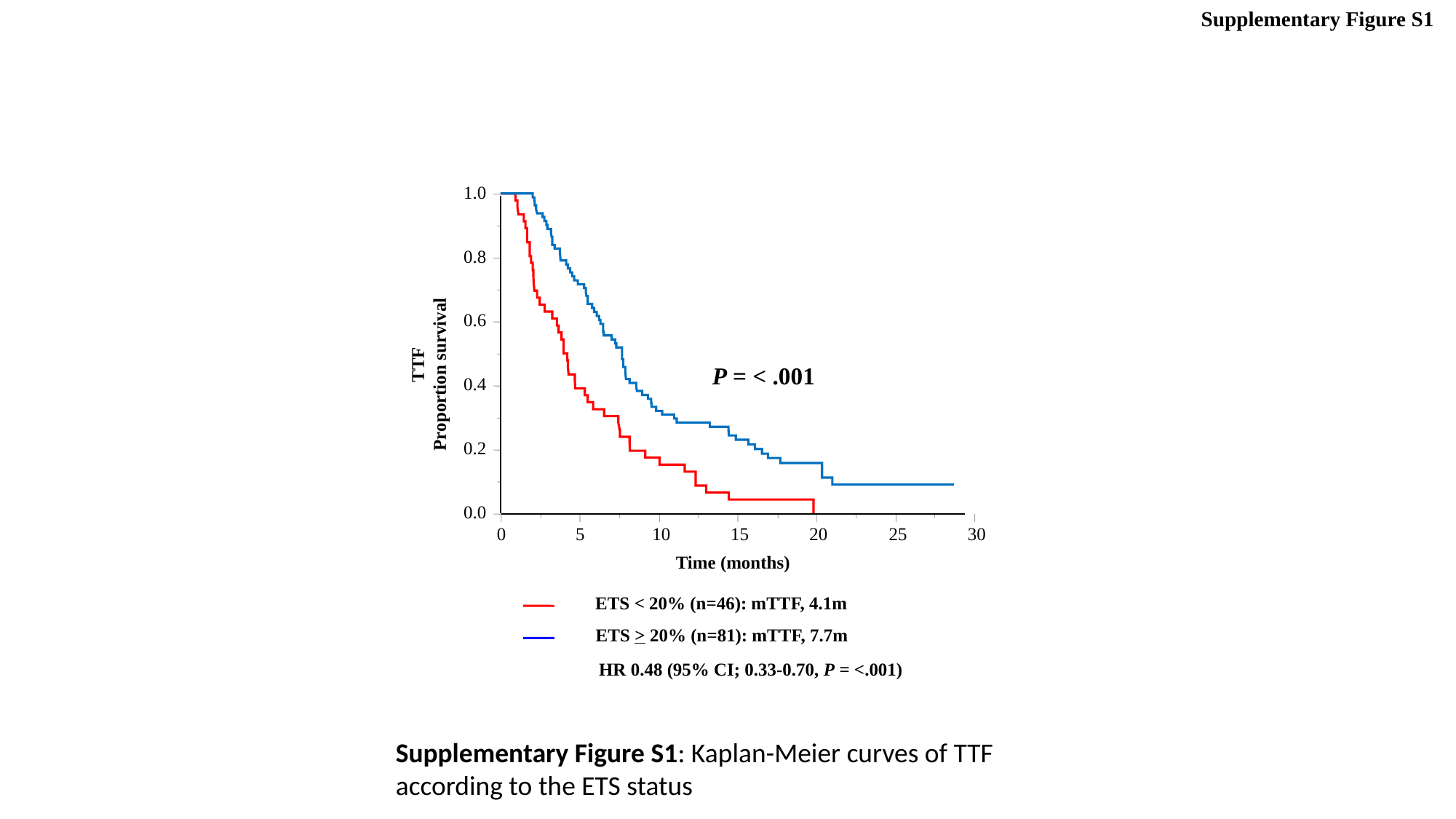

Supplementary Figure S1
1.0
0.8
0.6
0.4
0.2
0.0
0
5
10
15
20
25
30
 TTF
Proportion survival
P = < .001
Time (months)
ETS < 20% (n=46): mTTF, 4.1m
ETS > 20% (n=81): mTTF, 7.7m
 HR 0.48 (95% CI; 0.33-0.70, P = <.001)
Supplementary Figure S1: Kaplan-Meier curves of TTF according to the ETS status
