# Supplementary material for: Impact of early tumor shrinkage on quality of life in patients treated with first-line cetuximab plus chemotherapy for unresectable metastatic colorectal cancer: results of Phase II QUACK trial
Source: BMC Cancer. 2022 Jun 28;22:711. doi: 10.1186/s12885-022-09811-x (PMC9238042; doi:10.1186/s12885-022-09811-x)
Supplement: Supplementary file 2 — Additional file 2. [file 12885_2022_9811_MOESM2_ESM.pptx]

## Slide 1
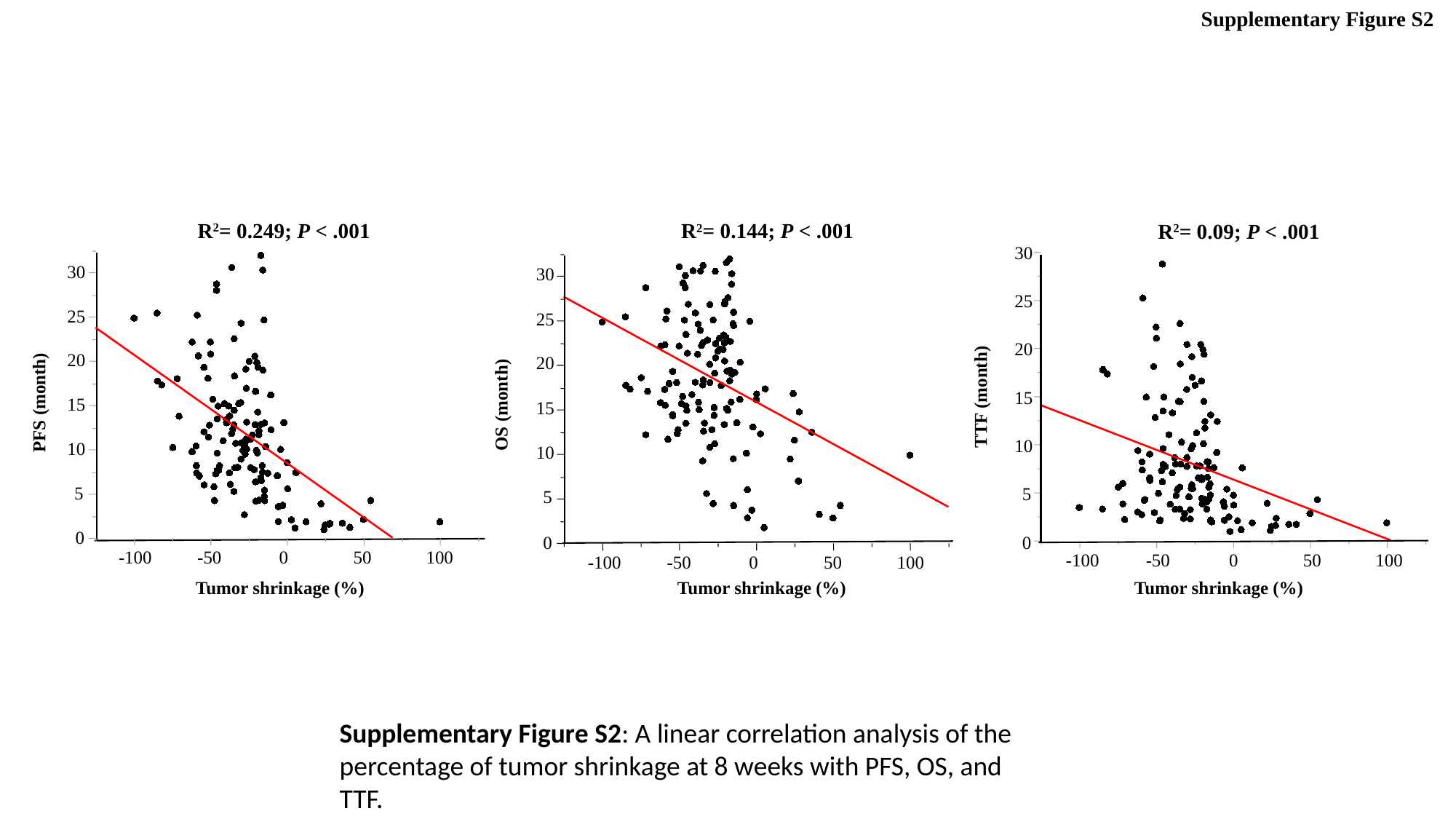

Supplementary Figure S2
R2= 0.09; P < .001
30
25
20
15
10
5
0
-100
-50
0
50
100
TTF (month)
Tumor shrinkage (%)
R2= 0.249; P < .001
30
25
20
15
10
5
0
-100
-50
0
50
100
PFS (month)
Tumor shrinkage (%)
R2= 0.144; P < .001
30
25
20
15
10
5
0
-100
-50
0
50
100
OS (month)
Tumor shrinkage (%)
Supplementary Figure S2: A linear correlation analysis of the percentage of tumor shrinkage at 8 weeks with PFS, OS, and TTF.
