# Supplementary material for: Impact of early tumor shrinkage on quality of life in patients treated with first-line cetuximab plus chemotherapy for unresectable metastatic colorectal cancer: results of Phase II QUACK trial
Source: BMC Cancer. 2022 Jun 28;22:711. doi: 10.1186/s12885-022-09811-x (PMC9238042; doi:10.1186/s12885-022-09811-x)
Supplement: Supplementary file 3 — Additional file 3. [file 12885_2022_9811_MOESM3_ESM.pptx]

## Slide 1
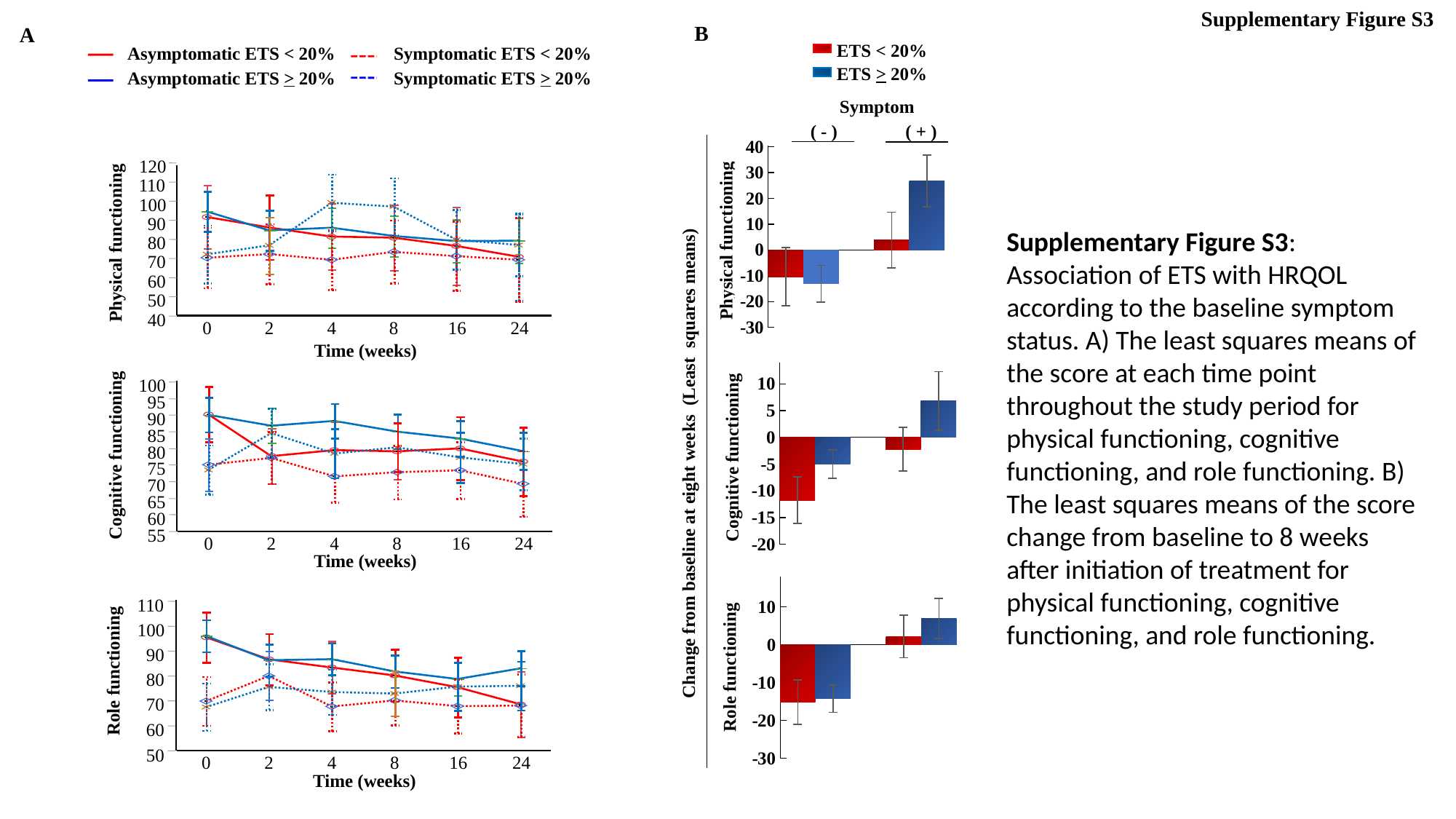

Supplementary Figure S3
B
A
ETS < 20%
ETS > 20%
Symptom
( - ) ( + )
 Symptomatic ETS < 20%
Asymptomatic ETS < 20%
Asymptomatic ETS > 20%
 Symptomatic ETS > 20%
### Chart
| Category | |
|---|---|Change from baseline at eight weeks (Least squares means)
120
110
100
90
80
70
60
50
40
0
2
4
8
16
24
Supplementary Figure S3: Association of ETS with HRQOL according to the baseline symptom status. A) The least squares means of the score at each time point throughout the study period for physical functioning, cognitive functioning, and role functioning. B) The least squares means of the score change from baseline to 8 weeks after initiation of treatment for physical functioning, cognitive functioning, and role functioning.
 Physical functioning
 Physical functioning
Time (weeks)
### Chart
| Category | |
|---|---|100
95
90
85
80
75
70
65
60
55
0
2
4
8
16
24
 Cognitive functioning
 Cognitive functioning
Time (weeks)
### Chart
| Category | |
|---|---|110
100
90
80
70
60
50
0
2
4
8
16
24
 Role functioning
 Role functioning
Time (weeks)
